# Modeling Caspian Sea water level oscillations under different scenarios of increasing atmospheric carbon dioxide concentrations

**DOI:** 10.1186/1735-2746-9-24

**Published:** 2012-12-12

**Authors:** GholamReza Roshan, Masumeh Moghbel, Stefan Grab

**Affiliations:** 1 Department of Geography, Golestan University, Gorgan, Iran; 2 Faculty of Geography, Department of Physical Geography, University of Tehran, Tehran, Iran; 3 School of Geography, Archaeology and Environmental Studies, University of the Witwatersrand, Johannesburg, South Africa

**Keywords:** Global warming, General circulation model, Carbon dioxide, Caspian sea water oscillations

## Abstract

The rapid rise of Caspian Sea water level (about 2.25 meters since 1978) has caused much concern to all five surrounding countries, primarily because flooding has destroyed or damaged buildings and other engineering structures, roads, beaches and farm lands in the coastal zone. Given that climate, and more specifically climate change, is a primary factor influencing oscillations in Caspian Sea water levels, the effect of different climate change scenarios on future Caspian Sea levels was simulated. Variations in environmental parameters such as temperature, precipitation, evaporation, atmospheric carbon dioxide and water level oscillations of the Caspian sea and surrounding regions, are considered for both past (1951-2006) and future (2025-2100) time frames. The output of the UKHADGEM general circulation model and five alternative scenarios including A1CAI, BIASF, BIMES WRE450 and WRE750 were extracted using the MAGICC SCENGEN Model software (version 5.3). The results suggest that the mean temperature of the Caspian Sea region (Bandar-E-Anzali monitoring site) has increased by ca. 0.17°C per decade under the impacts of atmospheric carbon dioxide changes (r=0.21). The Caspian Sea water level has increased by ca. +36cm per decade (r=0.82) between the years 1951-2006. Mean results from all modeled scenarios indicate that the temperature will increase by ca. 3.64°C and precipitation will decrease by ca. 10% (182 mm) over the Caspian Sea, whilst in the Volga river basin, temperatures are projected to increase by ca. 4.78°C and precipitation increase by ca. 12% (58 mm) by the year 2100. Finally, statistical modeling of the Caspian Sea water levels project future water level increases of between 86 cm and 163 cm by the years 2075 and 2100, respectively.

## Background

It is generally expected that increasing atmospheric concentrations of greenhouse gases, especially CO_2_, will lead to substantial global-scale climate changes over the next few decades. A probable consequence of these changes would be alterations in local and global sea level. Predictions of sea level change, especially sea level rise, are of considerable importance because of environmental and economic impacts, in particular on populations that inhabit low lying areas [1].

**Figure 1:**
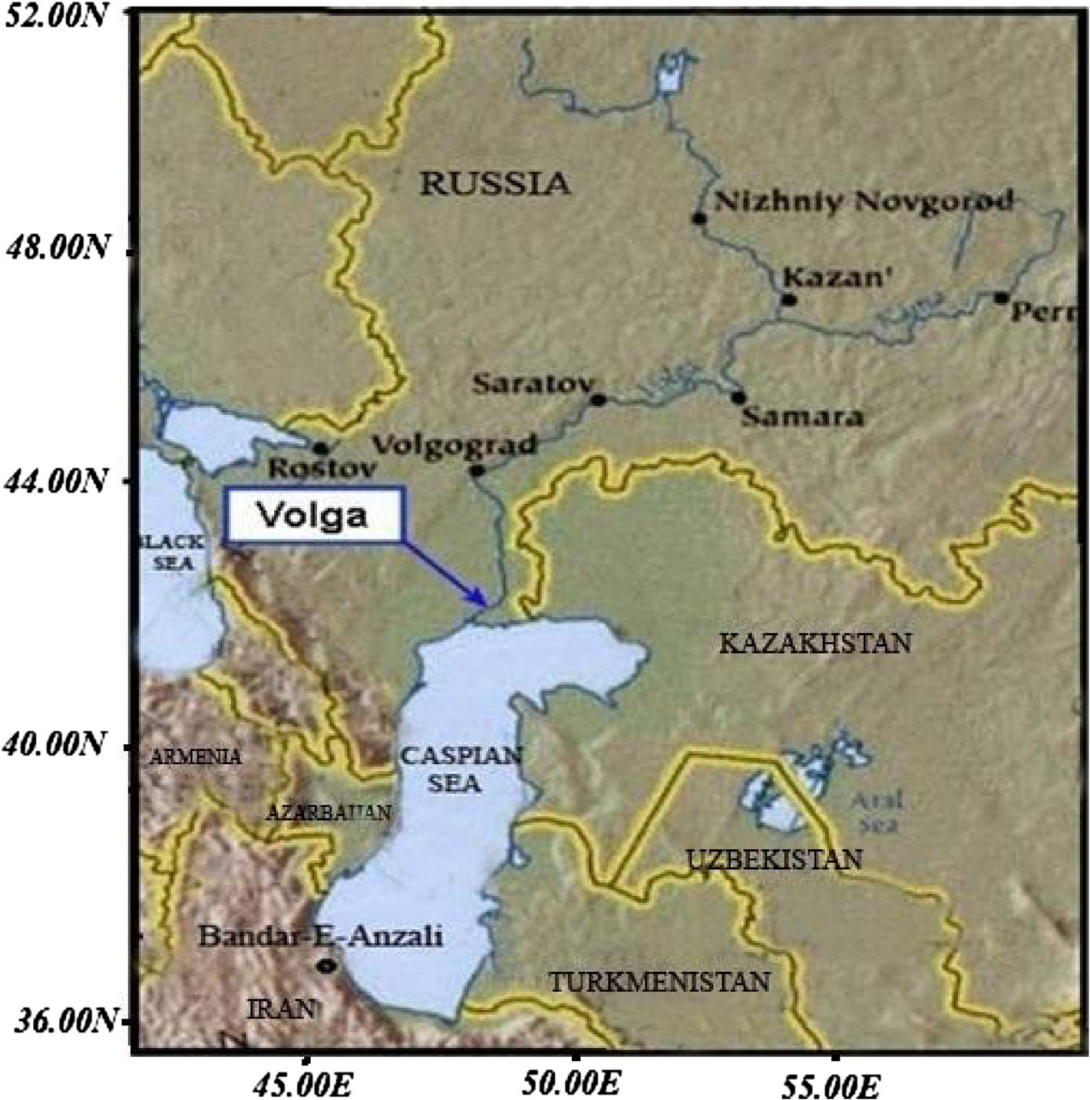
The Caspian Sea and Volga river water basins.

The Caspian Sea is the world’s largest lake which is located within the borders of five countries and thus serves as an important economic resource to these contries (Figure 1). The lake basin is generally divided into northern, mid and southern sections [2-4]. The Caspian Sea has existed for ca. 5.5 million years and accounts for 75% of the total volume of saline lakes. The lake is ca. 1200 km in length and averages 300 km in width. The Caspian Sea drainage basin extends from 30°N to about 62°N, covering an area of approximately 3.5 × 10^6^ km^2^. About 130 rivers flow into the sea, the largest of which is the Volga river which drains into the northern shelf of the Caspian Sea and accounts for approximately 80% of the total basin runoff.

The Caspian Sea is a closed water body which has a basin water budget and associated Caspian Sea level which is very responsive to inter-decadal climate fluctuations [5-8]. It is generally accepted that climate-induced changes in the hydrological budget of the Caspian Sea are the main cause of such CSL fluctuations. However, geological processes are also thought to influence the CSL, including tectonic movements [9,10] and deep groundwater flows between the Aral Sea and the Caspian Sea. In addition, anthropogenic activities such as land-use changes and reservoir developments have affected the CSL during the 20^th^ Century [11].

The Caspian Sea Level (CSL) has experienced considerable fluctuations during geological and historical times, and in particular, has risen 13 cm per year between 1977 and 1995, and continues to rise [12,13]. Historical observations and archaeological studies have revealed that the level of the Caspian Sea has varied by up to 8 m during the last 2 K years. The lowest recorded value occurred in 1977 at -29 m (i.e. below ocean level relative to that of the Baltic Sea). Since 1977, the sea level started to rise at an alarming rate, to a value of more than -27m [14,15]. Although recent past fluctuations of the lake have been calculated and discussed, little work has focused on current and future projected sea level changes. There are several important factors contributing to the oscillations of the Caspian Sea water level; one of the most important of these is regional climate change (warming), likely associated with increases in atmosphere carbon dioxide concentrations.

Even if there were a single country that surrounded the Caspian Sea, there would still be problems and tradeoffs in providing solutions related to the sea level rise, pollution, and resource development. However, because there are multiple countries involved, shared legacy, pollution and management issues, emergent highly profitable resources, divergent cultures, and debates over the scientific explanations for the sea level rise, there will continue to be environmental vulnerability associated with the Caspian Sea level rise [16,17]. Given that the water level changes in this sea have significant impacts on the environment, there have been numerous studies focusing on the Caspian Sea water level oscillations [12,18-30], and most of these have attempted to examine Caspian Sea level oscillations associated with climate changes and project future levels. The focus of our study is also to evaluate the impact of global warming, but more particularly to address a research gap by examining the role of associated CO_2_ levels on the CSL under different scenarios of Atmospheric Carbon Dioxide increases.

## Materials and methods

In order to gain better insight into the recent past relationships between Co_2_ and sea level fluctuations, temperature and precipitation data over the Caspian Sea region were analyzed over a 55 year period (1951-2006). To simulate future trends of Caspian Sea levels under global warming conditions, various climate change scenarios were generated for both the Caspian Sea region and Volga river basin using the MAGICC SENGENE model. Finally, Step Wise multiple regressions and SPSS (Statistical Package for the Social Sciences) software were used to model future Caspian Sea levels.

### Man-Kendall and linear regression methods

The Mann-Kendall test is a non-parametric test for identifying trends in time series data and compares the relative magnitudes of sampled data, rather than the data values. One benefit of this test is that the data do not need to conform to any particular distribution [31]. In this paper, the Mann-Kendall non-parametric test was used to access trends in temperature, precipitation and water level data for a 55 year period (1951-2006). A liner regression method is used for correlating temperature/precipitation and Caspian Sea water level oscillations. The Mauna Loa station Carbon Dioxide levels with Caspian Sea level oscillations was also correlated.

### Assessing the potential evaporation

A primary control on sea level oscillations is evapo-ration, a climatic parameter and process which is strongly driven by global warming, and indirectly by changes in CO_2_ concentrations [32]. The MAGICC SCENGEN model only simulates temperature and precipitation, and unlike models such as PENMAN and PENMAN MONITS, is unable to project future evaporation. Given that these evaporation models use different climate parameters and MAGICC SCENGEN is unable to simulate all of these parameters, the Torrens White model to simulate future evaporation over the Caspian Sea was used, as this model applies parameters such as temperature. The Thornthwaite model is processed as follows [33]:

•The thermal index is calculated from the following equation for every month of the year:

(1) $$i_{m}=\left(\frac{T_{m}}{5}\right)1.51$$

Where :

i_m=_Thermal index

T_m_ = Mean of monthly temperature in °C

•The annual thermal index is calculated from total monthly thermal index:

(2) $$I=\sum_{n=1}^{12}i_{m}$$

•Coefficient **a** is calculated from the following equation by using the annual thermal index:

(3) $$\begin{array}{c} a=\left(6.75\times 10^{-7}\right)I^{3}-\left(7.71\times 10^{-5}\right)I^{2}\\ \;+\left(1.792\times 10^{-2}\right)I+0.492 \end{array}$$

•Monthly evaporation values in mm are calculated from the following equation:

(4) $$\mathrm{PET}=16{\left(\frac{10T_{m}}{I}\right)}^{a}$$

### Climate change models

The Greenhouse-gas Induced Climate Change (MAGICC) model is used, together with a regional climate change database referred to as SCENario GENerator (SCENGEN) [34]. These models were developed at the Climate Research Unit, University of East Anglia, UK [35-38]. Grid data based on mean monthly, seasonal, and annual air temperatures and precipitation from 20 GCM experiments are available in the SCENGEN version 5.3 databases. The output data in MAGICC SCENGEN are displayed at a grid resolution of 2.5° latitude/longitude [39,40].

### Structure of the MAGICC SCENGEN model

The MAGICC model assesses gas-induced climate changes and is comprised of a set of simple interrelated models [37,38]. The model uses input parameters in the simulation process, the most important of which is climate sensitivity. The model is used for the purpose of simulating and projecting future climate scenarios for various regions. MAGICC is not a GCM model but uses data from some climate models to simulate GCM model behaviors for the considered region. The model incorporates a gas cycle as well as snow melt component, which permits the user to determine the average global temperature changes and the datum level changes according to greenhouse gas dispersions [37,38].

The principle of MAGICC/SCENGEN is to enable the user to explore the consequences of a wide range of future emission scenarios [39]. Projections are made based on modeled future scenarios of the dispersion of greenhouse gases; this is achieved by using different hypotheses in accordance to factors such as human activities, policies and technological applications. Using this method, 20 GCM models can be used separately or in groups. When selecting several GCM models, the program averages them and produces a compound model of climate change. However, in this project we only used the results of one GCM model (UKHADGEM) with 5 alternative scenarios (A1CAI, BIASF, BIMES WRE450 and WRE750) to analyze different conditions for the Caspian Sea region climate change scenarios.

## Results

### Relationship between Volga river discharge and Caspian Sea level oscillations

**Figure 2:**
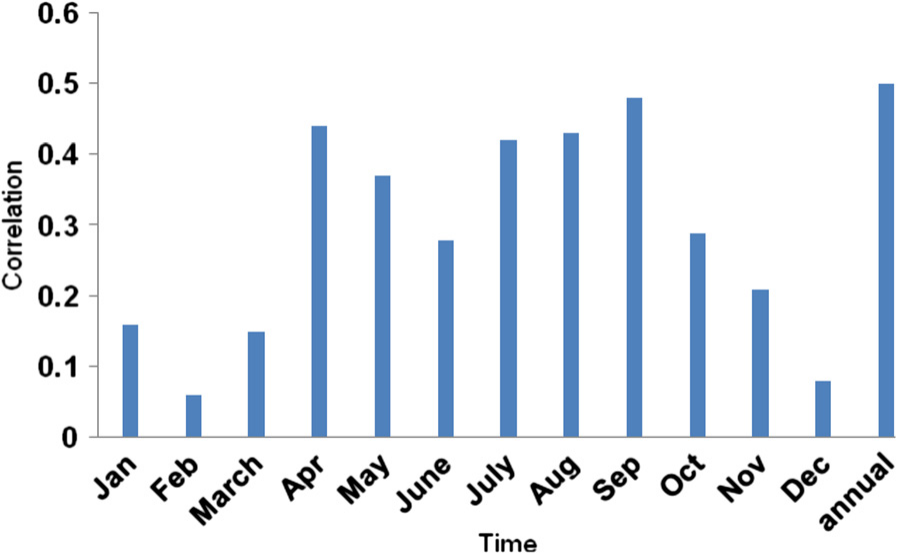
Monthly and annual correlation coefficient values reflecting the relationship between the Volga river discharge and Caspian Sea water level oscillations for the years 1951-2006.

The Volga river is the most important water supply to the Caspian Sea (80%), and thus the fluvial discharge has direct consequences on the Caspian Sea levels, so data for Volga discharge were extracted from the CRU (Climatic Research Unit) site and Caspian Sea level data were obtained from the Ports and Maritime Organization of the Islamic Republic of Iran. Monthly and annual correlations between the Volga River discharge and Caspian Sea levels for the statistical period 1951-2006 are presented in Figure 2. The mean annual correlation is r=0.47 (significance = 0.99); however, the relationship is weak during the cooler seasons from October through March (r=0.07; insignificance) but is substantially strengthened during the warmer seasons from April to September (r=0.44, significance=0.99). The Caspian sea levels thus rise during the warmer seasons (it increases by about 1 meter) when there is a greater volume of water entering the sea through the Volga river (volume average in summer is 415 km^3^), and subside during the colder seasons when the northern portion of the sea becomes frozen and the mean volume of Volga discharge decreases to ca. 40.1 km^3^; a likely consequence of frozen and snow-covered conditions in the upper drainage basin during these months.

### Impacts of carbon dioxide levels (based on the Mauna Loa station data) on Caspian Sea level oscillations

It is widely believed that there is a strong association between contemporary global warming and increased greenhouse gas concentrations (particularly CO_2_) in the atmosphere (e.g. [41-43]). The high elevation, free tropospheric site of Mauna Loa in Hawaii is reputed for its clean air status with ozone values similar to those of pre-industrial times [44]. To this end, Mauna Loa atmospheric records are regularly used for assessing global scale CO_2_ concentrations and changes within various site specific and global-scale environmental contexts (e.g. [45-47]). In this paper we compare the Mauna Loa CO_2_ records with surface temperature and evaporation levels, and water level oscillations from the Caspian Sea.

Correlations between temperatures (T_min_, T_max_, T_mean_) at Bandar-E-Anzali in the southwestern sector of the Caspian Sea, and CO_2_ concentrations at Mauna Loa do not indicate statistically significant relationships for the most part. However, the strongest relationship is recorded for T_min_ (r=0.27; significance =0.95). Minimum temperatures have increased at a rate of +0.45°C/decade at Bandar-E-Anzali, for the period 1951 to 2006. It is argued that T_min_ (nocturnal) trends should provide a better indication than T_max_ when determining the impact of greenhouse gases, as the influence of day-time radiation is eliminated [36]. Mean temperatures have increased at a rate of +0.17°C/decade at Bandar-E-Anzali, and a somewhat weaker positive correlation is recorded between T_mean_ and CO_2_ (r=0.21; significance =0.85). In contrast, T_max_ has decreased at a rate of -0.16°C/decade and demonstrates a statistically significant inverse relationship with CO_2_ concentrations (r= -0.37; significance l=0.98). Given that variations in discharge of the Volga River have important impacts on the water level and volume of the Caspian Sea, so the effect of atmospheric CO_2_ on climatic elements of the Volga River stations (Volgograd, Saratov, Samara, Kazan and Nizhny Novgorod) was also analyzed. Mean temperatures in the Volga river catchment have increased by +0.25°C/decade between the years 1951-2006 and there is a statistically significant correlation with changes in the Mauna Loa CO_2_ concentrations (r = 0.27; significance level=0.98). Results indicate weak correlations between variations in CO_2_ concentration and evaporation from the Caspian Sea surface (r=0.20) and Volga river (r=0.18). These results also indicate that evaporation from the Caspian Sea surface has increased at a rate of +7 mm/decade for the period 1951-2006, whilst it has increased by ca. +4 mm/decade in the Volga river basin. Finally, the strong positive correlation between Caspian Sea level variations and CO_2_ concentrations (r=0.82) demonstrates the strong influence that increasing CO_2_ concentrations have had on increasing the Caspian Sea levels between the years 1951-2006, at a rate of +36 cm/decade.

### Temperature and precipitation trends compared with Caspian Sea level variations during past decades

**Figure 3:**
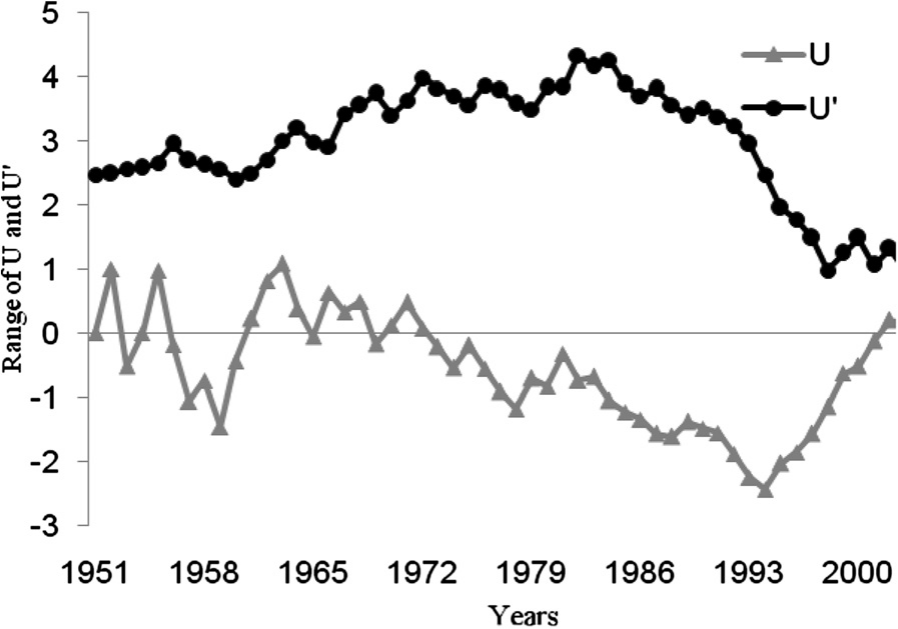
**Annual temperature trend in the southwestern Caspian Sea region during the past 55 years (1951-2006).** (U&U^′^: Man-Kendall Components. & U: actual trend for temperature. U^′^: inverse of the U).

**Figure 4:**
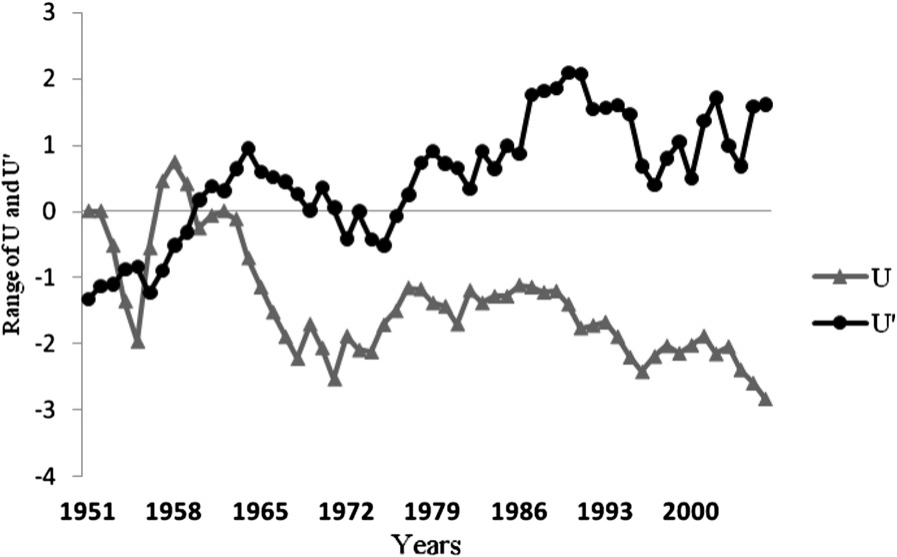
**Annual preceipitation trend in the southwestern Caspian Sea region during the past 55 years (1951-2006).** (U&U^′^: Man-Kendall Components. & U: actual trend for precipitation. U^′^: inverse of the U).

**Figure 5:**
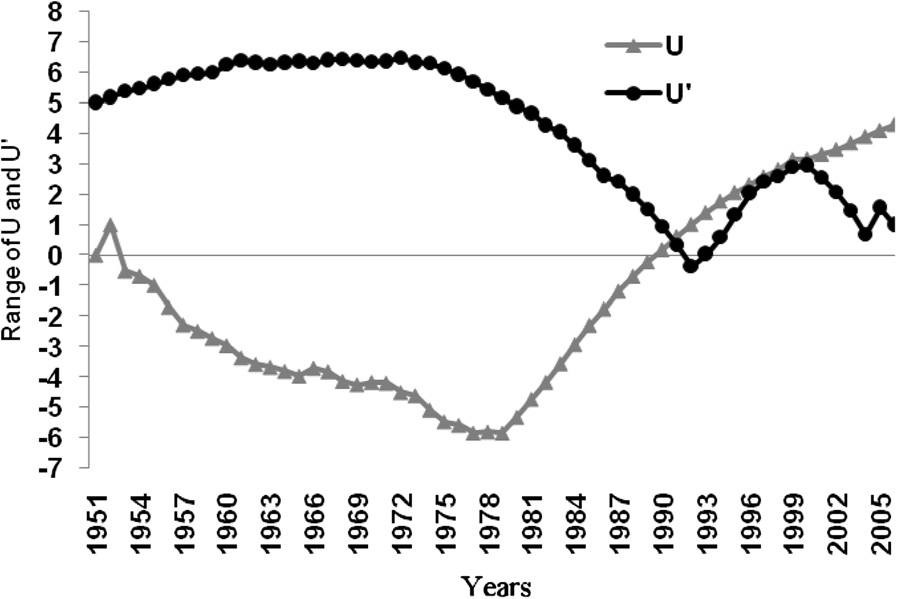
**Annual sea level trends in the southwestern Caspian Sea region during the past 55 years (1951-2006).** (U&U^′^: Man-Kendall Components. & U: actual trend for sea level. U^′^: inverse of the U).

Thermal properties of water typically have a lag-time in response to air temperatures; we thus used a one month delay when making comparisons (for example: temperature and precipitation values for August were correlated with water levels during September). The results indicate a correlation coefficient of r=0.26 when comparing annual precipitation and water levels, while that for mean annual temperature and water levels is r=0.30. Monthly values indicate that the highest correlation coefficient between precipitation and water level is measured in April (r=0.30, significance =0.98), whilst that for temperature and water levels is measured during August (r=0.41) (significance =0.99). It thus seems that there is a stronger relationship between temperature and water level oscillations of the Caspian Sea than between precipitation and water level oscillations (Table 1). However, according to the Man-Kendall method (Figures 3, 4, 5), temperatures seem to have risen substantially and provided for a stronger influence on water levels since 2000. It is important to note that given the greater variability of precipitation as opposed to temperature, it presents greater fluctuations according to the results of the Man-Kendall method and has had a decreasing trend and influence during recent years.

**Table 1:** Monthly and annual correlation coefficient of temperature and precipitation with water level oscillation at Bandar-E-Anzali station (1951-2006)

**Months**		**Jan**	**Feb**	**March**	**Apr**	**May**	**Jun**	**Jul**	**Auge**	**Sep**	**Oct**	**Nov**	**Dec**	**Annual**
Temperature	correlation	0.12	0.10	0.25	0.30	0.15	0.26	0.24	0.41	0.23	0.30	0.10	0.10	0.30
Precipitation		0.20	0.10	0.25	0.30	0.16	0.16	0.14	0.16	0.20	0.20	0.10	0.10	0.26

Time series source of climatic data (2010): Iran Meteorological Organization (IRIMO) [48]: http://www.irimet.net

### CO_2_ projections by 2100 using various modeling scenarios

**Figure 6:**
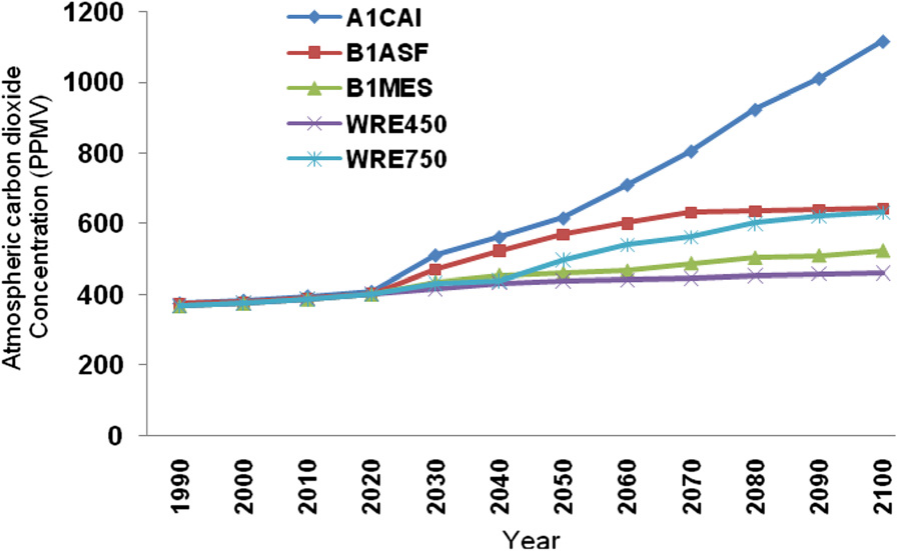
**Concentrations of CO**_**2 **_**projected for the year 2100 based on A1CAI, BIASF, BIMES, WRE450 and WRE750 scenarios.**

In order to project CO_2_ concentrations by 2100, five alternative scenarios suggested by the IPCC are used: A1CAI, BIASF, BIMES, WRE450 and WRE750. The A1 family is mainly based on increased education levels of families, increased investment rates and inventions in the fields of education, technology and energy at national and international levels. The B1 family is built on the premise of greater future environmental and social awareness with stabilized rates of development, whilst the WRE families are based on specific levels of atmospheric CO_2_. A more detailed review of these scenarios is presented by Roshan *et al.*[36]. When evaluating the simulated atmospheric CO_2_ concentrations according to various scenarios, the highest amount of CO_2_ is suggested by the A1CAI scenario, which would also be associated with highest temperature change projections (Figure 6). According to this scenario, carbon dioxide has a progressive increasing trend and is projected to reach 1100 ppm by the year 2100. In contrast, scenarios of the B1ASF and WRE families project similar trends of CO_2_, ranging between 450 ppm to 650 ppm by the year 2100.

### Projected Caspian Sea and Volga River temperature, evaporation and precipitation changes

**Figure 7:**
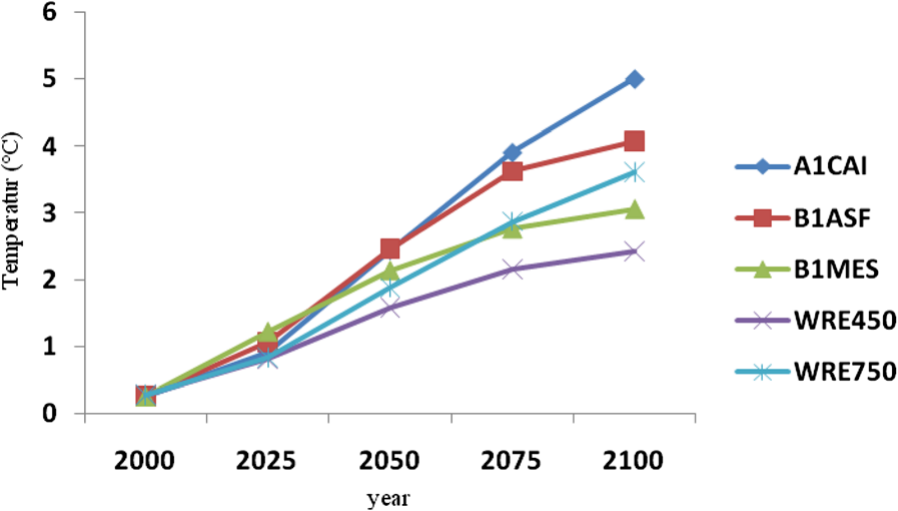
Projected temperature changes over the Caspian Sea for various years to 2100.

**Figure 8:**
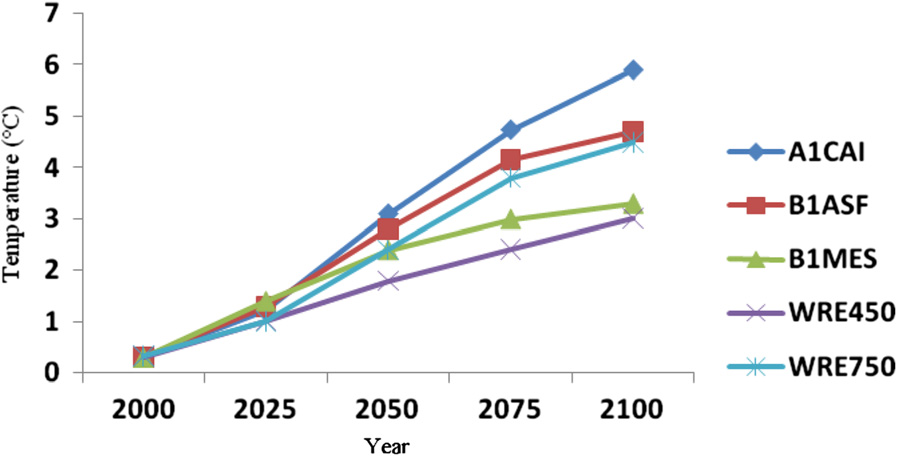
Projected temperature changes over the Volga River Basin for various years to 2100.

To evaluate temperature and precipitation changes in the Caspian Sea and Volga river basin, data from Bandar-E-Anzali and Volga stations were selected to first determine contemporary temperature, evaporation and precipitation characteristics, and then to compare these with those projected for future decades. As mentioned, five scenarios suggested by the IPCC are used to project such future temperature and precipitation changes, followed by calculations of rates of such changes based on various MAGICC SCENGEN model scenarios. Given that 80% of the water budget in the Caspian Sea is provided by the Volga River, we also evaluate the rate of temperature and precipitation changes for this river basin, which is performed on a monthly basis for five time intervals (2000-2025-2050-2075-2100), as is also undertaken for the Caspian Sea basin. Figure 7 and 8 provide the five scenarios based on the MAGICC SCENGEN model outputs.

Simulated temperature results indicate steadily rising projected trends for both the Caspian Sea and Volga river basin. The highest temperature increases are projected using the A1CAI scenario, according to which average of temperatures would rise by ca. 5°C and 6°C for the Caspian Sea and Volga river basin respectively by 2100, relative to the 1961-1990 reference period. The most optimistic result indicating lowest temperature increases is using the WRE450 scenario, with projected increases of 2°C and 3°C for the Caspian Sea and Volga River basin respectively by 2100. Thus, considerable differences in temperature increases are projected for the two basins, with average increases using all scenarios being 3.64°C for the Caspian Sea and 4.78°C for the Volga river basin by the year 2100 (Figures 7 and 8).

**Figure 9:**
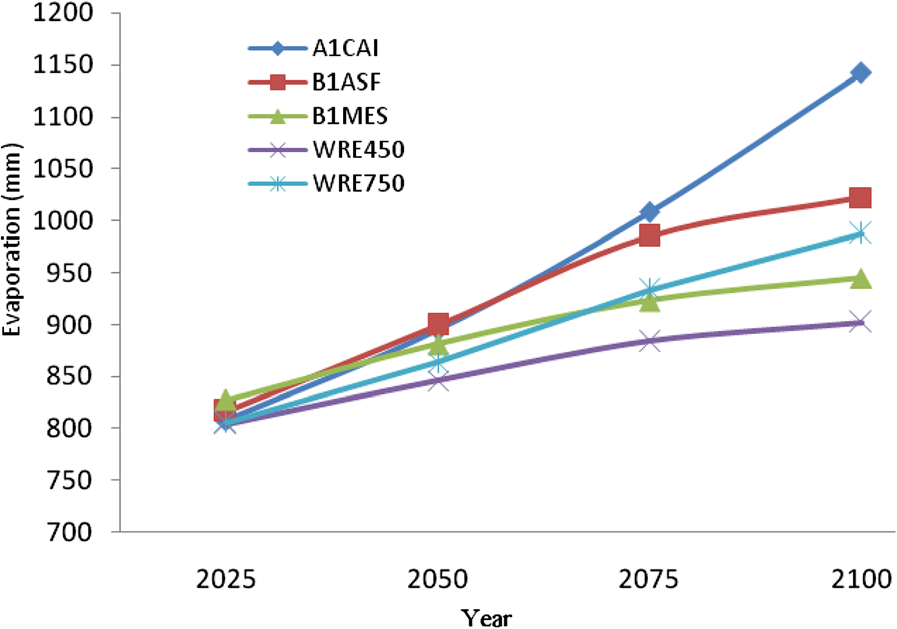
Projected increases in evaporation over the Caspian Sea for various years to 2100.

Calculated values of all simulated scenarios indicate that mean annual evaporation rates over the Caspian Sea surface will increase by ca. 133 mm by the year 2100, relative to that for the reference period 1961-1990. As with temperature, the most pessimistic outlook is using the A1CAI scenario which projects a ca. 350 mm increase in mean annual evaporation by 2100, whilst the most conservative increases are projected using the WRE450 scenario, with an increase of ca. 100 mm by the year 2100 (Figure 9).

**Figure 10:**
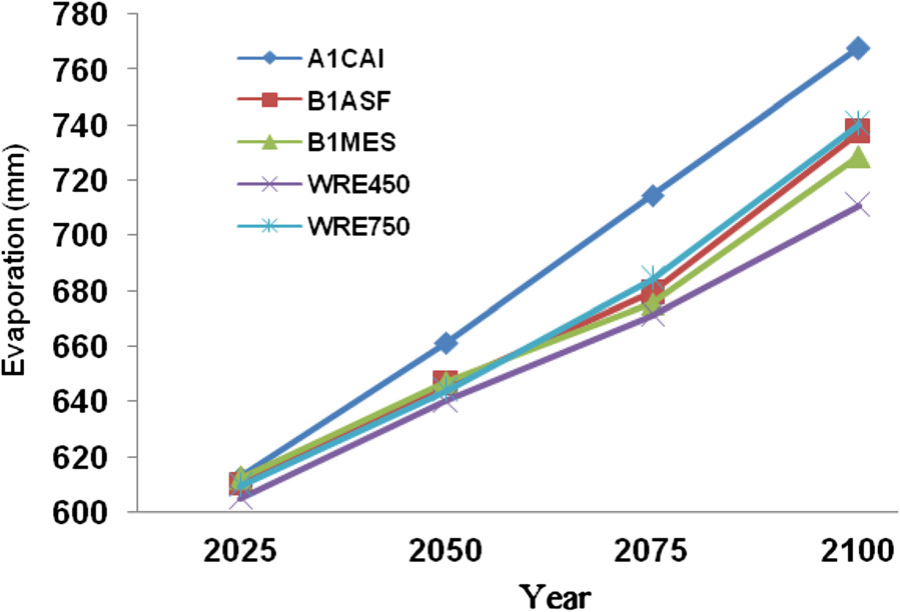
Projected increases in evaporation over the Volga River Basin for various years to 2100.

Considering future evaporation rates within the Volga river basin is equally important when modeling future Caspian Sea levels, given the primary role of this river’s discharge into the lake. Generally, projected evaporation rates for the Volga River Basin are highest for the A1CAI scenario, followed by the B and WRE scenarios (Figure 10). Mean projected annual evaporation increases relative to the 1961-1990 reference period are 122 mm by 2025, 160 mm by 2050, 197 mm by 2075 and 249 mm by the year 2100. These simulation results follow from a recent trend indicating that Caspian Sea surface evaporation rates have increased at a rate of 4.4 mm per decade over the period 1960-2006.

**Figure 11:**
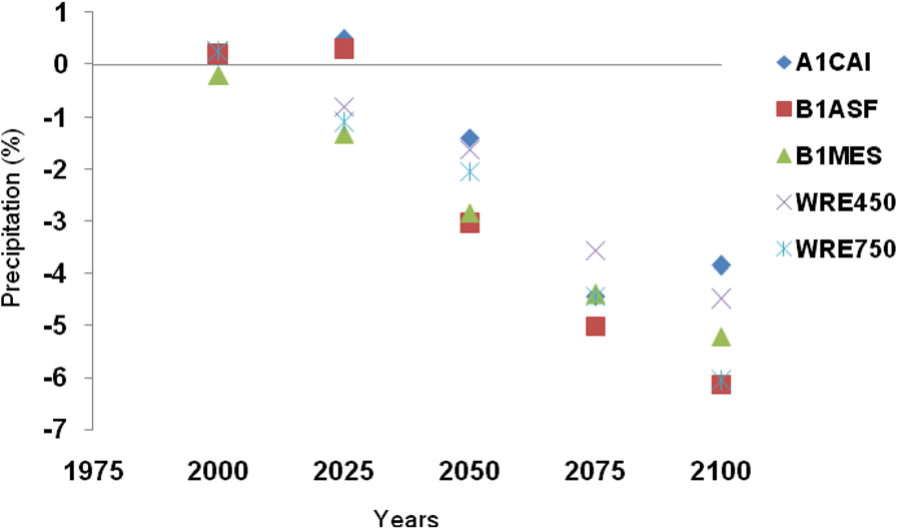
Projected precipitation changes over the Caspian Sea for various years to 2100.

**Figure 12:**
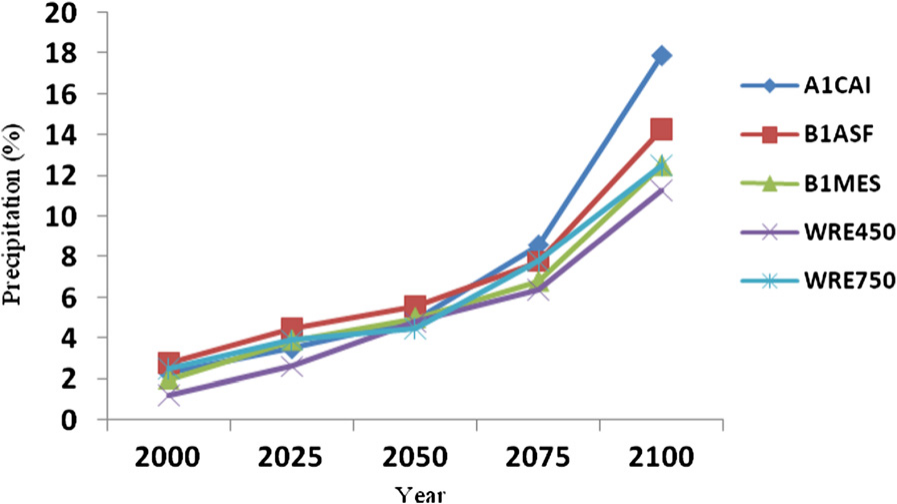
Projected precipitation changes over the Volga river basin for various years to 2100.

Precipitation is undisputedly a major driver determining river discharge and consequent lake levels. To this end, it is noteworthy that projected future precipitation trends follow an inverse relationship between that modeled for the Caspian Sea (decreasing trend) and that for the Volga river basin (increasing trend) relative to the 1961-1990 reference period (Figures 11 and 12). The mean projected precipitation increase for the Volga river basin is 12% (58 mm) by the year 2100, whilst that over the Caspian Sea is projected to decrease by 10% (182 mm) over the same period. The most pessimistic outlook is using WRE750 and WRE450 scenarios for the Caspian Sea and Volga River Basin respectively, whilst the most optimistic scenarios for both sub-regions is the A1CAI.

### Statistical modeling of projected future Caspian Sea level oscillations

**Figure 13:**
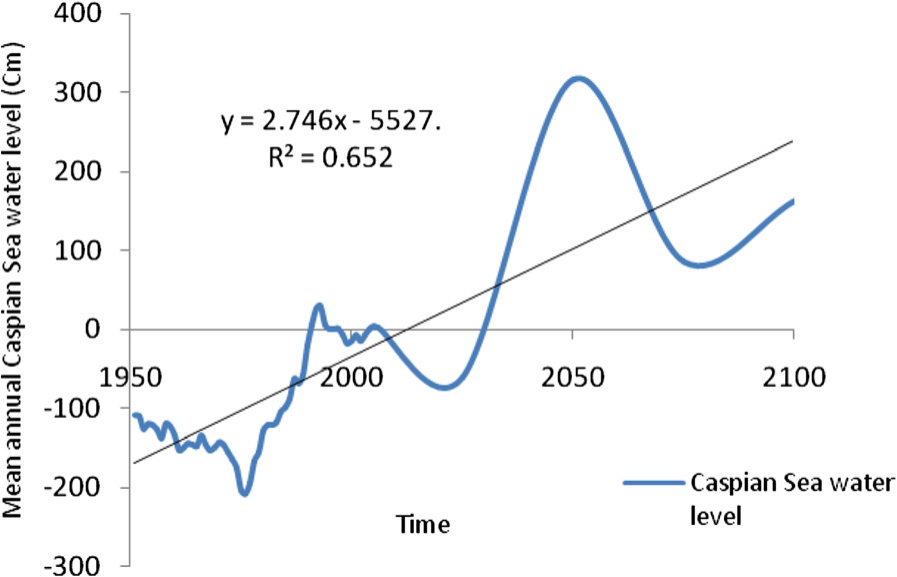
Modeled past and future Caspian Sea level oscillations.

A stepwise multiple regressions was used to determine which parameters best project future Caspian Sea water level fluctuations based on changes in CO_2_, temperature, precipitation and evaporation rates for the period 1951-2006. The results of this statistical model are then used as a basis upon which future Caspian Sea water levels are modeled. The analyses indicate that precipitation (Beta=-0.16, P=0.011), CO_2_ (Beta=0.92, P=0.000) and evaporation (Beta=-0.17, P=0.007) together explain 87% of the variance in Caspian Sea water level changes, whilst temperature (P=0.203) is not accounted for in the percentage variance in this process. After applying the Beta Coefficient on values of precipitation, evaporation and CO_2_ for the years 2025 to 2100, the results indicate an incremental increasing trend in sea level, such that the output of the regression model projects levels of -61.73, 316.22, 85.73 and 162.71 cm for the years 2025, 2050, 2075 and 2100, respectively. It is important to note here that the mean long-term Caspian Sea level change for the years 1961-1990 was an increase of 134.61 cm, but that a decrease of -61.73 cm is projected for the year 2025, after which levels will rise substantially until about the year 2050 (Figure 13).

It is likely that projected temperature increases over Russia and the Volga river basin will cause more moderate cold seasons than during the past decades, thus melting snow cover and increasing evaporation rates, which should consequently increase precipitation (most especially liquid precipitation) and lead to enhanced Volga river discharge. Such a process is likely to induce rising Caspian Sea water levels into future decades. Based on our modeled results, precipitation in the Volga river Basin is projected to increase by ca. 12% (58 mm) by the year 2100, and thus contribute considerably towards rising Caspian Sea levels. Rising water levels of the southern Caspian Sea (Iran) poses a variety of natural problems, thus the simulated values of projected rising sea levels (relatively conservative estimates) during future decades is something that must be planned towards, however worst case scenarios associated with future increasing discharge levels of the Volga River may require the consideration of less conservative estimates.

## Discussion

This paper examines the impact of CO_2_ increases on Caspian Sea water levels, which in turn influence a variaty of climate variables including temperature. Despite mean daily maximum temperatures having decreased by 0.16°C/decade (r=0.37), daily minumum and mean temperatures have increased at rates of 0.45°C/decade (r=0.27) and 0.17°C/decade (r=0.21), respectively for the southern Caspian Sea region of Bandar-E-Anzali for the period 1951-2006. In addition, evaporation levels have increased at a rate of 7 mm/decade (r=0.24) during the same statistical period. Results for the Volga River Basin, which is the primary hydrological input to the Caspian Sea, indicate a mean temperature increase of 0.25°C/decade (r=0.27) and rate of evaporation increase of 4.4 mm/decade (r=0.18) for the period 1951-2006.

The Caspian Sea water level has increased at a rate of 36 cm/decade (r=0.82) in relation to CO_2_ and temperature increases from the 1950s to 2000s. To simulate future climate scenarios the UKHADGEM model through the MAGICC SCENGEN software was used, which includes the application of five alternative scenarios using A1CAI, BIASF, BIMES, WRE450 and WRE750. The most pessimistic projection outcomes are associated with A1CAI (CO_2_ = 1100 ppm) whilst the most optimistic are using WRE scenarios (CO_2_ = 450 ppm) by the year 2100. Further to this, the highest projected temperature increases for the Caspian Sea and Volga river basin are 5°C and 6°C respectively, using the A1CAI scenario, whilst the lowest increases are projected using the WRE450 scenario (2°C and 3°C respectively). Mean projected temperature increases for the Caspian Sea and Volga River Basin are 3.46°C and 4.78°C respectively by the year 2100. Mean projected precipitation values are calculated to decrease by 10% (182 mm) over the Caspian Sea and increase by 12% (58 mm) in Volga River Basin by the year 2100. Based on statistical modeling, we project Caspian Sea levels to increase by 85.73 cm and 162.71 cm by the years 2075 and 2100 respectively.

## Conclusion

It is anticipated that major cities adjacent to the Caspian Sea, such as Anzali and Ramsar amongst others, need to plan for such sea level rises which would be accompanied by the flooding of agricultural land, possible structural damage in the coastal zone through wave erosion, and changes in the coastal zone ecology. It is suggested that any planned construction and development in the current coastal zone needs to heed to the warning that water levels are likely to continue rising for the next few decades to come.

## Competing interests

The authors declare that they have no competing interests.

## Authors’ contributions

GHR carried out data gathering and analyzing, assisting in the writing, drafted and submission of the manuscript. MM has made running the model, assisting in the writing, drafted and submission of the manuscript. GS has been involved in drafting the manuscript, assisting in climatic data preparation of the Volga Basin and revising it critically. All authors read and approved the final manuscript.
